# Modulation of the Immune-Inflammatory Microenvironment by Implant Material Properties in Peri-Implantitis

**DOI:** 10.3390/ijms27073006

**Published:** 2026-03-26

**Authors:** Siyu Liu, Guangjie Chen

**Affiliations:** Department of Immunology and Microbiology, Shanghai Jiao Tong University School of Medicine, Shanghai 200025, China; sjtulsy888@sjtu.edu.cn

**Keywords:** peri-implantitis, dental implants, immune-inflammatory microenvironment

## Abstract

Dental implant technology is widely used in clinical practice as a key approach for restoring partially or fully edentulous jaws. However, the occurrence of peri-implantitis significantly impacts the long-term success rate of dental implants, making it one of the key challenges in the field of implant dentistry. Peri-implantitis involves pathological changes in both soft and hard tissues surrounding dental implants, with its core pathological mechanism closely associated with the dysregulation of the immune inflammatory microenvironment. This article systematically reviews the core components of the immune-inflammatory microenvironment in peri-implantitis, including the activation mechanisms and functions of macrophages, the complement system, Langerhans cells, and adaptive immune cells, as well as the roles of key molecular pathways in regulating this microenvironment. It further explores the interaction mechanisms between implant material properties and the immune-inflammatory microenvironment. Finally, it summarizes current prevention and treatment strategies and provides an outlook on future research directions in this field.

## 1. Introduction

Dental implant technology has become a crucial approach for restoring partially or fully edentulous jaws, owing to its excellent biocompatibility, functional efficacy, and esthetic outcomes. Since the 1960s, dental implant technology has achieved significant advancements in materials, techniques, and implant design. However, implant failure remains a primary concern for both dentists and patients. Peri-implantitis, as one of its most common biological complications, has an average prevalence rate of 19.53% [1] and is one of the leading causes of implant failure [2].

Peri-implantitis is a bacterial inflammation occurring in the peri-implant tissues, primarily characterized by inflammation of the soft tissues and bone loss around the implant [3], which impairs the formation of osseointegration following implant placement. Implant loosening and failure due to peri-implantitis can impair chewing function, thereby affecting food intake and nutrient absorption. Furthermore, implant failure necessitates retreatment, which not only prolongs treatment duration but also imposes a financial burden on patients. Elucidating the pathogenic mechanisms and risk factors of peri-implantitis is therefore crucial for its prevention and treatment. The immune-inflammatory microenvironment around the implant plays a significant role in the progression of peri-implantitis by regulating bone regeneration and remodeling.

The immune-inflammatory microenvironment refers to the dynamic microenvironment formed by immune cells, tissue cells, inflammatory mediators, and extracellular matrix under pathological conditions such as tissue injury or infection. Within peri-implant tissues, an imbalance in this immune-inflammatory microenvironment may arise from suboptimal implant material properties, persistent microbial challenge, or aberrant host immune responses, ultimately leading to peri-implantitis. This review focuses on the role of implant material properties in modulating the immune microenvironment in peri-implantitis.

## 2. Core Components and Activation Mechanisms of the Immune-Inflammatory Microenvironment

**Figure 1:**
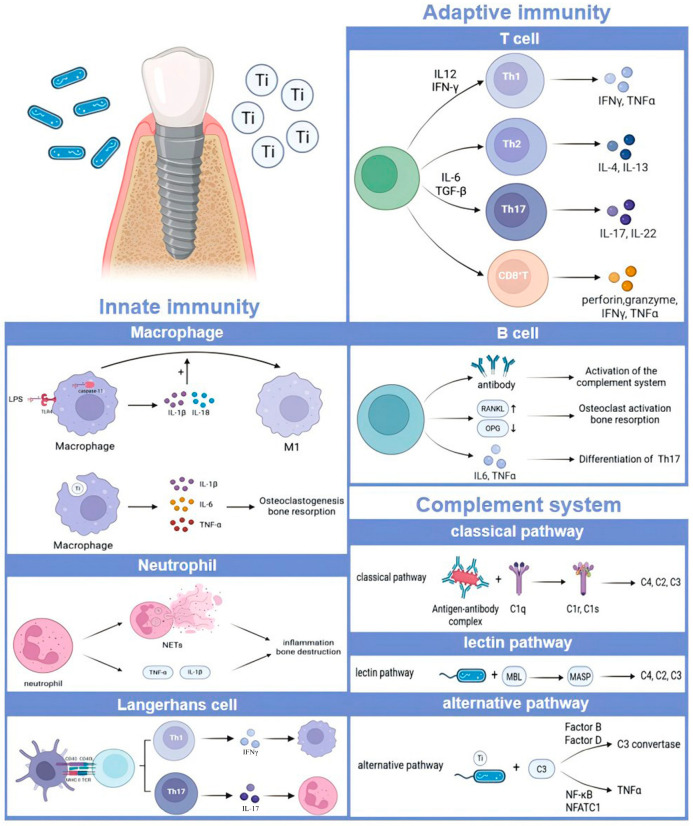
Core components and activation mechanisms of the immune-inflammatory microenvironment, including the innate immunity, the adaptive immunity and the complement system.

The immune-inflammatory microenvironment arises from dynamic interactions between innate immune cells, adaptive immune effectors, and the complement system. These components collectively detect threats, coordinate inflammatory responses, and regulate tissue repair or damage. As illustrated in Figure 1, their activation mechanisms are tightly interlinked: innate immunity provides rapid initial defense, adaptive immunity delivers antigen-specific precision, and the complement system bridges both while amplifying inflammation. Below, we detail their roles and crosstalk in shaping immune-inflammatory outcomes.

### 2.1. Innate Immune Cells: Functions and Activation Mechanisms

In peri-implant tissues, the primary cells involved in the innate immune response include macrophages, neutrophils, and Langerhans cells. Their functional activation is closely associated with the onset and progression of peri-implantitis.

#### 2.1.1. Macrophages

Macrophages play a crucial regulatory role in the process of pathological bone resorption around implants, being associated with inflammatory cell recruitment, increased numbers of mature osteoclasts, and the secretion of inflammatory cytokines.

Macrophages exhibit high plasticity and can polarize into pro-inflammatory M1 macrophages or anti-inflammatory M2 macrophages under the regulation of different signals. The M1/M2 macrophage polarization trend is closely related to the foreign body reaction around implants [4]. In peri-implant tissues, the polarization and activation of macrophages are regulated by multiple signaling pathways, among which the Nuclear Factor κB (NF-κB) pathway serves as a core regulatory mechanism [5]. Pathogenic LPS binds to Toll-like receptor 4 (TLR4) on the macrophage surface while simultaneously interacting with the intracellular pattern recognition receptor caspase-11. This interaction further triggers inflammasome activation, leading to the massive release of IL-1β and IL-18, thereby promoting M1 macrophage polarization [6]. Furthermore, macrophages can phagocytose titanium ions and release cytokines such as IL-6, IL-1β, and TNF-α, which in turn induce osteoclastogenesis and bone resorption [7].

#### 2.1.2. Neutrophils

Neutrophils are the first innate immune cells recruited to the peri-implant tissues following implant placement. Their primary functions are to eliminate pathogens and necrotic tissue, playing a critical role in the initiation of the inflammatory response.

During the early stages of peri-implantitis, appropriate recruitment and activation of neutrophils can clear bacteria and control the spread of inflammation. However, as inflammation persists and progresses, activated neutrophils continuously infiltrate peri-implant tissues, releasing neutrophil extracellular traps (NETs) and large amounts of pro-inflammatory mediators such as TNF-α and IL-1β, which trigger inflammation and bone destruction [8]. NETs can capture and neutralize microorganisms via their mesh-like structures, preventing the spread of microbial infection. However, this process also entraps cellular debris and bacteria, making them difficult to clear and leading to sustained neutrophil activation. Furthermore, excessive NETs release substantial amounts of histones, which upregulate the IL-17/Th17 response and promote bone destruction, exacerbating the inflammatory reaction [9].

#### 2.1.3. Langerhans Cells

Langerhans cells are primarily located in the basal and spinous layers of the gingival epithelium surrounding dental implants. As a subtype of dendritic cells, they serve as the major antigen-presenting cells (APC) in oral epithelium. Studies indicate that titanium implants can impair the development of human oral Langerhans cells, potentially leading to immune dysregulation [10].

In the early phase of peri-implantitis, Langerhans cells become activated upon uptake of bacterial antigens and present these antigens, thereby activating a limited number of T cells and initiating a mild adaptive immune response. However, persistent antigen stimulation leads to massive activation of Langerhans cells, which in turn activate more T cells. For example, Th1 cells secrete IFN-γ, further promoting M1 macrophage polarization and exacerbating the inflammatory response [11]. Th17 cells secrete IL-17, promoting neutrophil recruitment and forming a NETs-Th17 positive feedback loop [12], ultimately accelerating bone resorption.

### 2.2. Adaptive Immune Cells and Their Functions and Activation Mechanisms

#### 2.2.1. T Cells

Studies indicate that exposure to titanium materials can promote T cell activation and differentiation toward inflammatory subsets, increasing the secretion of corresponding cytokines [13]. The T cells involved in the immune-inflammatory microenvironment around implants primarily include Th1, Th2, Th17, and CD8^+^ T cells.

Th1 cells are mainly induced and differentiated by IL-12 and IFN-γ, highly expressing the transcription factor T-bet and secreting pro-inflammatory cytokines such as IFN-γ and TNF-α [14]. Th2 cells secrete IL-4 and IL-13, which promote M2 macrophage polarization [15]. IL-4 enhances osteoblast activity by the JAK/STAT pathway, thereby contributing to tissue repair [16]. Th17 cells are primarily differentiated under the induction of IL-6 and TGF-β, secreting cytokines like IL-17 and IL-22 to promote bone resorption around implants [17]. Upon activation, CD8^+^ T cells release perforin and granzymes to directly kill bacteria-infected cells around implants, reducing the spread of infection. They also secrete pro-inflammatory factors such as IFN-γ and TNF-α, synergizing with Th1 cells to enhance inflammatory response [18].

#### 2.2.2. B Cells

B cells participate in peri-implant inflammatory responses and bone metabolism regulation by secreting antibodies and expressing immunomodulatory molecules in response to antigen stimulation. The antibodies secreted by B cells can bind to antigens, forming antigen–antibody complexes that activate the complement system. Concurrently, the Fc portion of antibodies can bind to Fc receptors on the surface of macrophages, enhancing the phagocytic capacity of macrophages against bacteria or titanium particles.

In terms of immune regulation, B cells can modulate bone metabolism by expressing Receptor Activator of NF-κB Ligand (RANKL) and osteoprotegerin (OPG). Studies indicate that activated B cells exhibit upregulation of RANKL expression and downregulation of OPG expression on their surfaces, which promotes osteoclast activation and bone resorption [19]. Furthermore, pro-inflammatory cytokines such as IL-6 and TNF-α secreted by B cells can enhance Th17 cell differentiation, further exacerbating the inflammatory response.

### 2.3. Mechanisms of Complement System Activation and Functions

#### 2.3.1. Mechanisms of Complement System Activation

In peri-implantitis, the complement system can be activated through the classical pathway, the lectin pathway, and the alternative pathway. The classical pathway is primarily initiated by antigen–antibody complexes. These complexes bind to complement C1q, activating C1r and C1s, which in turn sequentially activate C4, C2, and C3, thereby initiating the classical pathway [20]. The lectin pathway is mainly triggered by mannose residues on bacterial surfaces. Bacteria colonizing the implant surfaces, such as Porphyromonas gingivalis, are rich in mannose residues. Mannose-binding lectin (MBL) recognizes and binds these mannose residues, activating MBL-associated serine protease (MASP). MASP subsequently activates C4, C2, and C3 components, initiating the lectin pathway [21]. The alternative pathway is primarily activated by foreign particles on the implant surface or bacterial cell wall components. Titanium particles and bacterial LPS on the implant surface [22] can directly activate complement C3. The presence of Factor B and Factor D leads to the formation of the C3 convertase, which subsequently activates downstream complement components and initiates the alternative pathway. C3 can stimulate the secretion of pro-inflammatory factors such as TNF-α through the NF-κB pathway and Nuclear Factor of Activated T-cells 1 (NFATC1), leading to peri-implantitis [22].

#### 2.3.2. Functions of Complement System

The complement system plays multiple roles in the immune-inflammatory microenvironment of peri-implant tissues. C3a and C5a are potent chemokines that bind to C3aR and C5aR on the surface of macrophages and neutrophils, inducing the recruitment of immune cells to the site of peri-implantitis. Additionally, C3a can activate platelets, promoting platelet aggregation and the release of coagulation factors, which initiates the coagulation process [23]. Research has revealed that the concentration of C5a in the gingival crevicular fluid of patients with peri-implantitis is significantly higher than that in the healthy implant group. Moreover, C5a levels show a positive correlation with both probing depth and bone resorption, suggesting its key role in immune cell recruitment and disease progression [24]. C3b and C4b can covalently bind to the surface of bacteria or foreign particles, forming opsonins. The C3b receptor (CR1) on neutrophils recognizes C3b, enhancing the phagocytosis of opsonized bacteria or particles [25]. The terminal product of complement system activation is the membrane attack complex (MAC). MAC assembles on the membrane of bacterial or infected cells to form pores that lyse pathogens, leading to intracellular osmotic imbalance and ultimately causing the lysis and death of the target cells [26].

## 3. Key Molecular Pathways

The homeostasis of the peri-implant immune-inflammatory microenvironment relies on the precise regulation of multiple molecular pathways. Among these, the chemokine network, the RANKL/RANK/OPG pathway, and ferroptosis-related pathways serve as core regulatory mechanisms that integrate the functions of immune cells, tissue cells, and inflammatory mediators, directly influencing the progression of the lesion.

### 3.1. Chemokine Network

In peri-implantitis, the primary chemokine families involved are CXC and CC. Key CXC chemokines include CXCL8 and CXCL10. CXCL8 serves as a chemokine specific to neutrophils. It is secreted by macrophages and epithelial cells upon stimulation by LPS or TNF-α and induces the recruitment of neutrophils to the lesion site [27]. IFN-γ secreted by Th1 cells can induce the secretion of CXCL10, which plays a significant role in the migration of monocytes and activated T cells [28]. Prominent CC chemokines include CCL5. CCL5 is secreted by T cells and monocytes and, by binding to CCR5 expressed on T cells, smooth muscle cells, and endothelial cells, plays a role in processes such as cell proliferation, angiogenesis, and inflammation [29].

### 3.2. RANKL/RANK/OPG Pathway

The RANKL/RANK/OPG pathway is a core pathway regulating osteoclast differentiation and bone resorption, linking immune inflammatory responses with bone metabolism. Its equilibrium directly determines the stability of peri-implant bone tissue.

RANKL is primarily secreted by T cells (such as Th17 cells), B cells, and osteoblasts and functions as a type II transmembrane protein. RANK is expressed on the surface of osteoclast precursors and mature osteoclasts, serving as the specific receptor for RANKL. OPG, secreted by osteoblasts and vascular endothelial cells, acts as a soluble antagonist of RANKL by binding to it and preventing its interaction with RANK [19]. The binding of RANKL to RANK triggers the activation of Tumor Necrosis Factor Receptor-Associated Factor 6 (TRAF6) within osteoclasts, activating the NF-κB pathway and MAPK pathways (such as JNK and p38). This leads to the upregulation of osteoclast-specific genes, including Tartrate-Resistant Acid Phosphatase (TRAP) and Cathepsin K, ultimately inducing osteoclast differentiation and activation [30].

In peri-implantitis, multiple factors can elevate the RANKL/OPG ratio, disrupting the homeostasis of the RANKL/RANK/OPG pathway. For example, IL-17, secreted by Th17 cells, can stimulate RANKL production from osteoblasts and T cells while suppressing OPG expression [31]; LPS can activate the TLR4 pathway in osteoblasts, promoting RANKL secretion and reducing OPG production [32]; titanium particles can be phagocytosed by macrophages, activating NLRP3 inflammasomes to promote IL-1β release, thereby upregulating RANKL expression and exacerbating bone resorption [33].

### 3.3. Ferroptosis-Related Pathways

Ferroptosis is a novel form of regulated cell death characterized by the iron-dependent accumulation of lipid peroxides, ultimately leading to cell death. Recent studies have revealed that ferroptosis-related pathways play a significant role in the peri-implant immune inflammatory microenvironment. These pathways can influence disease progression by regulating immune cell functions and the survival of bone cells.

Ferroptosis primarily depends on three key events: iron ion accumulation, lipid peroxidation, and GPX4 functional inhibition. In peri-implantitis, titanium particles or LPS can induce intracellular iron accumulation and downregulation of GPX4 expression in macrophages, leading to ferroptosis in these cells [34]. Macrophages undergoing ferroptosis release large amounts of damage-associated molecular patterns (DAMPs), such as high-mobility group box 1 (HMGB1) and ATP. These DAMPs can further activate macrophages and neutrophils, amplifying the inflammatory response [35].

## 4. Interaction Between Implant Material Properties and the Immune-Inflammatory Microenvironment Around the Implant

After being implanted into the body as a foreign material, the properties of a dental implant—such as its surface morphology, chemical composition, and degradation behavior—directly interact with the host immune system. This interaction modulates the homeostasis of the immune-inflammatory microenvironment, thereby influencing the occurrence of peri-implantitis. Therefore, gaining a deep understanding of the interaction mechanisms between implant material properties and the immune-inflammatory microenvironment is crucial for optimizing implant design and improving implant success rates.

### 4.1. Surface Morphology

#### 4.1.1. Roughness

Implant surfaces with different roughness exhibit significant differences in their regulatory effects on the immune-inflammatory microenvironment. Ultra-smooth surfaces exhibit weaker protein adsorption capacity and a limited contact area with immune cells, which impedes the activation of mechanosensory signals. Studies indicate that ultra-smooth surfaces reduce the number of early-stage adhering macrophages, inhibit macrophage polarization toward the M1 type, and decrease secretion levels of TNF-α and IL-1β [36]. However, excessively smooth surfaces are unfavorable for osteoblast adhesion and proliferation. Bacteria can form biofilms on such ultra-smooth surfaces, and prolonged stimulation may still trigger chronic inflammation, compromising osseointegration stability.

The microscopically rough surfaces (e.g., titanium surfaces treated by sandblasting and acid etching (SLA)) are currently the most widely used implant surface morphology in clinical practice. It features a micro-scale structure of pits and protrusions and can maintain the stability of the immune-inflammatory microenvironment through the following mechanisms: First, by regulating the spreading morphology of macrophages (e.g., promoting the formation of multipolar pseudopodia), it activates mechanosensitive pathways within the cells, thereby inducing M2 polarization of macrophages [37]. Second, by enhancing the hydrophilicity of titanium materials, selective adsorption of serum proteins is achieved, prioritizing the adsorption of osteointegration-promoting proteins such as fibronectin while reducing the adsorption of pro-inflammatory proteins and the release of pro-inflammatory factors, thereby balancing immune regulation with osteointegration efficiency [38].

Regarding the mechanisms underlying material-cell interactions, implant surface roughness can achieve targeted regulation of cellular functions by modulating the mechanosensory receptors of immune cells and osteoblasts. A study comparing the effects of smooth titanium surfaces and modified rough titanium surfaces on integrin expression and cytokine secretion in M0/M1 macrophages confirmed that titanium surface roughness can regulate the expression levels of integrins α2, αM, and β1, thereby influencing the secretion of pro-inflammatory cytokines such as IL-1β, TNF-α, and IL-31 by macrophages, providing experimental evidence for the regulation of immune cell function by mechanical signals [39].

#### 4.1.2. Topological Structure

In addition to roughness, the topological structure of the implant surface can also influence the peri-implant immune-inflammatory microenvironment. Implant surfaces with directional groove structures (e.g., micro-scale grooves prepared via laser etching) can guide the oriented alignment of macrophages. Studies have found that groove structures can promote macrophage spreading along the groove direction, suppressing cellular overactivation and reducing the secretion of pro-inflammatory factors [40]. Porous structures can promote bone tissue ingrowth by increasing implant-bone contact area, while their pore architecture can regulate the infiltration and function of macrophages [41]. Nanostructured arrays (e.g., titanium nanotubes, nanopillars) can modulate immune cell activation through nanoscale surface features. For example, in vitro studies demonstrate that specific nanoscale topological structures enhance macrophage adhesion and spreading, promoting their polarization toward the M2 phenotype. The construction of anisotropic TiO_2_ nanoporous structures on titanium surfaces via anodization confirms that such nanoscale topological structures facilitate macrophage cytoskeletal reorganization and activate the PI3K-AKT signaling pathway, thereby enhancing macrophage adhesion and spreading, inducing M2 polarization, and increasing VEGF secretion [42].

#### 4.1.3. Surface Charge Characteristics and Electrostatic Interactions

The surface topography of implant materials modulates protein adsorption and cell adhesion by altering surface charge distribution and mediating interfacial electrostatic interactions. For instance, an ultra-smooth titanium surface exhibits a uniform charge distribution. Under physiological conditions at approximately pH 7.4, negatively charged albumin and positively charged lysozyme adsorb onto the ultra-smooth titanium surface via electrostatic interactions, with the adsorbed amount increasing with time and applied electrical potential [43].

### 4.2. Chemical Composition

The chemical composition of implant materials directly influences their biocompatibility and degradation properties. Among these, titanium and titanium alloys, along with zirconia, are the two most commonly used implant materials in clinical practice today.

#### 4.2.1. Titanium and Titanium Alloys

Titanium and its alloys are the most widely used implant materials in clinical practice due to their excellent biocompatibility, corrosion resistance, and mechanical properties. They exhibit minimal interference with the immune-inflammatory microenvironment. When exposed to air or within the body, titanium and titanium alloys form a dense oxide layer (primarily composed of TiO_2_). This oxide layer possesses good chemical stability, preventing the release of metal ions and reducing stimulation to the immune system. Additionally, the TiO_2_ oxide layer exhibits hydrophilic properties, which promotes the adsorption of anti-inflammatory proteins (e.g., albumin) from serum while inhibiting the adsorption of pro-inflammatory proteins (e.g., fibrinogen) [44].

#### 4.2.2. Zirconia

Zirconia (ZrO_2_) is a ceramic implant material whose regulatory effects on the immune-inflammatory microenvironment differ from those of titanium and titanium alloys. The zirconia surface possesses high surface energy and hydrophilicity, which promote osteoblast adhesion and differentiation [45]. Additionally, it resists bacterial colonization, thereby reducing bacterial biofilm formation.

Compared to titanium, zirconia surfaces adsorb fewer pro-inflammatory proteins and exhibit a lower degree of macrophage activation [46]. Furthermore, zirconia exhibits an extremely slow degradation rate in vivo, releasing only trace amounts of Zr^4+^ ions. Notably, Zr^4+^ ions demonstrate no significant cytotoxicity or immunostimulatory effects, thereby avoiding the induction of marked inflammatory responses [47].

#### 4.2.3. Molecular Mechanisms of Implant Material–Cell Interactions

For the TiO_2_ oxide film on titanium and titanium alloys, as well as the high-surface-energy hydrophilic structure of zirconia, the underlying mechanism involves the interaction of integrin β1 with adsorbed fibronectin (FN), which directly influences macrophage polarization toward the M2 phenotype. In contrast, macrophages adhered to hydrophobic surfaces interact with adsorbed fibrinogen (FG) via integrin β2, leading to M1-type macrophage polarization. Furthermore, integrin β1 has been observed to drive macrophage polarization toward the M2 phenotype through the PI3K/AKT signaling pathway, whereas integrin β2 promotes M1-type macrophage polarization via NF-κB activation. Therefore, approaches that modulate macrophage behavior based on surface hydrophilicity not only enhance our understanding of macrophage responses but also hold significant promise for the design of immunomodulatory implants [48].

### 4.3. Degradation Behavior

The degradation behavior of implant materials in vivo (including degradation rate and the type and concentration of degradation products) is a key factor influencing the long-term stability of the immune-inflammatory microenvironment. Even for materials with good biocompatibility, uncontrolled degradation can induce sustained immune stimulation and lead to peri-implantitis.

Products released during implant material degradation (e.g., metal ions and particulate fragments) can trigger inflammatory responses through the following mechanisms: Micron- or nano-scale particles generated during material degradation can be phagocytosed by macrophages. If these particles cannot be degraded by lysosomes, they accumulate within macrophages, leading to their activation and fusion into foreign body giant cells. These foreign body giant cells can cause local tissue damage and chronic inflammation by releasing pro-inflammatory factors such as TNF-α and IL-1β. High concentrations of degradation products exhibit cytotoxicity and can induce apoptosis or necrosis in macrophages and osteoblasts. Cell death releases DAMPs (e.g., HMGB1, ATP), which further activate surrounding immune cells and disrupt the balance of the immune-inflammatory microenvironment [49]. Meanwhile, titanium particles can induce peri-implantitis by activating the NLRP3 inflammasome while concurrently inhibiting the GSK-3β/β-catenin pathway in osteoblasts, leading to suppressed osteogenesis and enhanced bone resorption [50].

## 5. Strategies for Preventing and Treating Peri-Implantitis Based on Implant Material Properties

### 5.1. Surface Morphology Optimization

Optimizing implant surface morphology represents a promising strategy for the prevention of peri-implantitis. The core rationale is to precisely design micro- or nano-scale topological features to balance moderate activation of immune cells with functional induction of osteoblasts, thereby avoiding excessive inflammatory responses and osseointegration failure. Combined with bioactive coatings or nanostructural modifications, this approach further enhances the surface’s anti-inflammatory properties and osteocompatibility; it is also one of the areas with the highest potential for clinical translation in current implant material research and development. In vitro studies have shown that osteoblasts cultured on nano-hydroxyapatite (nHA) surfaces exhibit superior spreading compared to those cultured on other surfaces, while simultaneously reducing the Bax/Bcl2 ratio to inhibit apoptosis [51]. In terms of in vivo testing, animal studies have demonstrated that implants coated with nHA exhibit superior bone repair outcomes in rat tibial models, effectively mitigating local bone resorption while exhibiting a low rate of nHA loss [52]. In clinical studies, head-to-head comparisons have shown that nHA-coated implants demonstrate superior immediate stability during the early osseointegration phase compared to uncoated SLA implants [53], suggesting that constructing nHA coatings on a microscopically rough surface can regulate the peri-implant immune-inflammatory microenvironment through the bioactivity and nano-effects of nHA.

Nano-array structures, such as titanium nanotubes, represent another important approach to surface modification, and significant progress has been made in their in vivo research and clinical translation. In vivo animal studies have further revealed that titanium nanotubes (particularly those with a diameter of 70 nm) can modulate bone formation at the bone-implant interface, inducing beneficial molecular responses at the genetic level and thereby promoting osseointegration [54].

In addition, composite surfaces modified with nHA and titanium nanotubes have become a hot topic of research in recent years. Studies have shown that when TiO_2_ nanotubes are used as a bridging system between titanium alloys and thin layers of hydroxyapatite nanocrystals, this composite material exhibits excellent properties. It outperforms other nanocomposites studied in terms of fibroblast adhesion and proliferation, while also demonstrating effective antibacterial activity. Furthermore, this system features high surface roughness, an appropriate elastic modulus, and resistance to plastic deformation, making it an important direction for future optimization of implant surface topography [55].

### 5.2. Chemical Component Regulation

Optimizing the composition design of implant materials to reduce the release of toxic ions and enhance anti-inflammatory properties can lower the risk of peri-implantitis. In traditional titanium alloys such as Ti-6Al-4V, the long-term release of aluminum and vanadium can induce immunotoxicity. Compositional substitution and alloying design can significantly improve their immune compatibility. Research indicates that the Ti-28Nb-5Zr-2Ta-2Sn (TNZTS) alloy exhibits a significantly reduced elastic modulus while maintaining comparable in vitro biocompatibility. Furthermore, TNZTS alloy specimens did not induce cytokine release from macrophages, suggesting its potential as a substitute for Ti-6Al-4V [56].

Concurrently, incorporating antimicrobial elements like Ag into titanium alloys enables antibacterial and anti-inflammatory effects through ion release. Studies reveal that Ti-Ag alloys (2 wt% Ag) can slowly release Ag^+^ ions in simulated body fluids, effectively inhibiting bacterial growth. This superior antimicrobial capability persists for at least 30 days [57].

### 5.3. Functional Modification

In addition to the physicochemical surface treatments described above, the construction of functional coatings loaded with immunomodulatory active molecules on implant surfaces has emerged as one of the key strategies for regulating the peri-implant immune-inflammatory microenvironment. Such coatings enable the localized and controlled release of anti-inflammatory or immunomodulatory factors, thereby suppressing excessive inflammatory responses while promoting tissue integration and bone repair. For instance, low-pressure plasma (LPP) and atmospheric pressure plasma (APP) treatments of titanium dental implants can downregulate the gene expression of pro-inflammatory cytokines, including IL-1β and TNF-α, which may be beneficial for the early osseointegration process [58].

The trace element selenium (Se) is an essential micronutrient for humans and plays a critical role in antioxidant defense and immune regulation. Studies have shown that loading selenium nanoparticles onto SLA-treated titanium implant surfaces effectively reduces oxidative stress levels in macrophages, inhibits activation of the NF-κB signaling pathway, decreases the secretion of pro-inflammatory cytokines such as TNF-α and IL-1β, and induces macrophage polarization toward the M2 phenotype, thereby establishing a local immune microenvironment favorable for anti-inflammatory responses and osteogenesis [59].

Glucocorticoids, such as dexamethasone and prednisone, are classic potent anti-inflammatory agents widely used to control excessive immune responses. Loading dexamethasone onto implant surfaces can reduce peri-implant inflammation by inhibiting activation of the NF-κB signaling pathway in macrophages, increasing the expression of IL-10 and TGF-β genes while decreasing the expression of TNF-α and IL-1 genes [60]. However, the release of glucocorticoids must be tightly controlled to avoid adverse effects on osteoblast differentiation and bone formation.

The construction of functional coatings can be achieved through various approaches, including plasma treatment and chemical grafting, providing a versatile platform for precisely modulating local immune responses. Collectively, integrating immunomodulatory active molecules into implant surface coatings represents a key direction in current implant material research. This strategy complements optimization approaches targeting surface topography and chemical composition, collectively offering the potential to achieve an ideal balance between immune regulation and osseointegration.

## 6. Conclusions

Peri-implantitis poses a critical challenge to the long-term success of dental implant therapy. Its pathogenesis is fundamentally rooted in the dysregulation of the peri-implant immune-inflammatory microenvironment. Inappropriate design of implant surface topography can trigger excessive activation of immune cells. The immunostimulatory nature of chemical components or uncontrolled degradation behavior can lead to the persistent release of inflammatory stimuli. These factors ultimately disrupt the implant-bone interface and induce bone resorption through abnormal immune cell responses and dysregulated molecular pathways. Consequently, focusing on the intrinsic properties of implant materials and precisely modulating their interaction with the immune-inflammatory microenvironment emerges as a pivotal strategy for preventing and managing peri-implantitis.

Although this review systematically summarizes the key mechanisms of the immune-inflammatory microenvironment in peri-implantitis and the preventive strategies targeting implant material properties, many novel materials and material modification techniques have demonstrated significant potential in basic research and preclinical studies. However, several limitations remain. First, most existing studies are based on animal models or in vitro experiments, which differ to some extent from clinical realities. Second, the synergistic mechanisms among different immune components have yet to be fully elucidated. Third, research on the impact of individual variability on material–immune interactions remains insufficient. Furthermore, the immunomodulatory effects of most novel materials currently lack validation from long-term clinical follow-up data. Addressing these critical issues will drive further advancement in this field.

Looking ahead, the deep integration of materials science and immunology holds promise for further optimizing the immunomodulatory capabilities of implant materials. This advancement will not only deliver more efficient and safer solutions for peri-implantitis but also propel the field of dental implantology towards greater precision and functionality. It will also reduce the risk of implant failure and ensure long-term osseointegration and oral health by precisely regulating the inflammatory microenvironment around the implant, ultimately offering a superior therapeutic experience for patients with partially or fully edentulous jaws.

## Data Availability

No new data were created or analyzed in this review. Data sharing is not applicable to this article.

## Abbreviations

The following abbreviations are used in this manuscript:

NF-κB	Nuclear Factor κB
TLR4	Toll-like receptor 4
NETs	neutrophil extracellular traps
APC	antigen-presenting cells
JAK	Janus kinase
STAT	Signal transducer and activator of transcription
RANKL	Receptor Activator of Nuclear Factor-κB Ligand
OPG	osteoprotegerin
MBL	Mannose-binding lectin
MASP	MBL-associated serine protease
NFATC1	Nuclear Factor of Activated T-cells 1
MAC	membrane attack complex
RANK	Receptor Activator of Nuclear Factor-κB
TRAP	Tartrate-Resistant Acid Phosphatase
DAMPs	damage-associated molecular patterns
HMGB1	high-mobility group box 1
SLA	sandblasting and acid etching
nHA	nano-hydroxyapatite
TNZTS	Ti-28Nb-5Zr-2Ta-2Sn
LPP	low-pressure plasma
APP	atmospheric pressure plasma
